# Analysis of facial bone fractures: An 11-year study of 2,094 patients

**DOI:** 10.4103/0970-0358.63959

**Published:** 2010

**Authors:** Kun Hwang, Sun Hye You

**Affiliations:** 1 Department of Plastic Surgery, Inha University College of Medicine, Incheon, Korea; 2 Center for Advanced Medical Education by BK21 Project, Inha University College of Medicine, Incheon, Korea

**Keywords:** Facial bones, facial fractures, injury prevention, treatment

## Abstract

**Purpose:**

The medical records of these patients were reviewed and analysed to determine the clinical characteristics and treatment of facial bone fractures.

**Patients and Methods:**

This is a retrospective study of 2,094 patients with facial bone fractures from various accidents that were treated at the Inha University Hospital from 1996 to 2007.

**Results:**

The most common age group was the third decade of life (29%). Males were more common than females (3.98:1). The most common aetiology was violent assault or nonviolent traumatic injury (49.4%). The most common isolated fracture site was the nasal bone (37.7%), followed by the mandible (30%), orbital bones (7.6%), zygoma (5.7%), maxilla (1.3%) and the frontal bone (0.3%). The largest group with complex fractures included the inferior region of the orbital floor and zygomaticomaxilla (14%). Closed reduction was performed in 46.3% of the cases while 39.7% of the cases required open reduction. For open reductions, the most commonly used soft-tissue approach was the intraoral approach (32.3%). The complication rate was 6.4% and the most common complication was hypoesthesia (68.4%) followed by diplopia (25.6%).

**Conclusion:**

Long-term collection of epidemiological data regarding facial fractures and concomitant injuries is important for the evaluation of existing preventive measures and useful in the development of new methods of injury prevention and treatment.

## INTRODUCTION

Facial injuries occur in a significant proportion of trauma patients requiring prompt diagnosis of fractures and soft tissue injuries, with possible emergency interventions.[1] Each year, increasing numbers of patients are admitted to the hospital with facial trauma.[1] There are many studies in the literature that have analysed the demographic factors associated with facial trauma according to various criteria.[2‐4] The epidemiology of facial fractures varies with regard to injury type, severity and cause, depending on the population studied.[5] The differences in the populations with regard to the causes of facial fractures may be the result of differences in culture and a variety of risk factors. However, the reports on patients studied, use the severity of the injury as the major selection criteria for epidemiological investigation.[5]

An understanding of the cause, severity and temporal distribution of facial trauma can aid in establishing clinical and research priorities for effective treatment and prevention of these injuries.[5] Continuous long-term collection of data regarding the epidemiology of facial fractures is important because it provides information necessary for the development and evaluation of preventive measures that might help reduce the incidence of facial injuries.[5]

The purpose of this study was to report on the natural history of facial injuries in 2,094 cases over 11 years at the Department of Plastic and Reconstructive Surgery, Inha University Hospital, Incheon, South Korea.

## MATERIAL AND METHODS

The medical records of patients seeking treatment for facial trauma were reviewed at the Department of Plastic and Reconstructive Surgery, Inha University Hospital, Incheon, South Korea. The study population consisted of 2,094 severely injured patients with facial fractures from February 1996 to April 2007, who were admitted to our hospital for operation and conservative treatment. The parameters assessed were age, gender, time of injury, aetiology and associated injuries, in addition to the type of fracture and treatment offered. The facial bone fractures were classified as isolated or complex fractures. The isolated facial bone fractures included frontal bone, orbital bone, nasal bone, maxilla, zygoma and mandible. The complex fractures were subclassified into five types according to the anatomical direction from the orbit and the extension from an adjacent area, which were superior, medial, lateral, inferior or other locations [Table 1]. In addition, the complications and prognoses were analysed.

**Table 1:** Classification of facial bone fractures

*Fracture*	*Types*	*Locations*	*Cases*	*%*
Isolated			1,720	82.1
	Frontal bone		7	0.3
	Orbital bone		159	7.6
		Floor	95	4.5
		Medial wall	42	2.0
		Roof	4	0.2
		Lateral wall	3	0.1
		Combined	15	0.7
	Nasal bone		790	37.7
	Maxilla		27	1.3
	Zygoma		119	5.7
		Zygomatic arch	77	3.7
		Zygomaticomaxilla	42	2.0
		Zygomaticofrontal bone	0	0
	Mandible		618	30.0
		Angle	149	7.1
		Symphysis or parasymphysis	143	6.8
		Condyle	70	3.3
		Body	26	1.2
		Ramus	3	0.1
		Combined	227	10.8
Complex			374	17.9
	Superior		6	0.2
		Frontal and orbital roof	1	0
		Frontal, orbital roof and nasal bone	5	0.2
	Medial		16	0.8
		Orbit medial wall and nasal bone	16	0.8
	Lateral		4	0.2
		Orbit lateral wall and zygomatic arch	4	0.2
	Inferior		293	14.0
		Zygomaticomaxilla and orbital floor	66	3.2
		Zygomaticomaxilla, orbital floor and nasal bone	42	2.0
		Orbital floor and nasal bone	17	0.8
		Tripod	130	6.2
		Zygomatic and nasal bone	38	1.8
	Others		55	2.7

## RESULTS

### Demographic distribution

This retrospective study of 2,094 cases included 1,673 males and 421 females, aged 1–97 years (mean age = 30.6 years) with facial bone fractures [Table 2]. The highest frequency of facial bone fractures was in the age group 21–30 years (*n* = 608, 29%), followed by 11–20 years (*n* = 466, 22.3%) and 31–40 years (*n* = 439, 21%) [Table 2]. There was a significant male predominance in all age groups and the overall ratio of males to females was 3.98:1.

**Table 2:** Distribution according to age and gender

*Age*	*Male*	*Female*	*Cases (%)*
0–10	64	31	95 (4.5)
11–20	396	70	466 (22.3)
21–30	188	120	608 (29.0)
31–40	349	90	439 (21.0)
41–50	222	73	295 (14.1)
51–60	94	21	115 (5.5)
>60	60	16	76 (3.6)
Total cases (%)	1,673 (79.9)	421 (20.1)	2,094 (100)

The analysis of the annual incidence revealed that both the absolute number and the proportion of facial injuries peaked in 2006 [Table 3], with slight increases every year [Table 3]. The monthly incidence was relatively even; however, facial fractures were slightly more common during the month of September (*n* = 209, 10%) [Table 4].

**Table 3:** Annual incidence

*Year*	*Cases*	*%*
1996. 2 ~	64	3.1
1997	163	7.8
1998	155	7.4
1999	156	7.5
2000	177	8.5
2001	242	11.6
2002	219	10.5
2003	214	10.2
2004	184	8.8
2005	217	10.4
2006	271	12.9
~ 2007. 4	32	1.5
Total	2,094	100

**Table 4:** Monthly distribution

*Month*	*Cases*	*%*
1	148	7.1
2	142	6.8
3	172	8.2
4	161	7.7
5	191	9.1
6	179	8.5
7	169	8.1
8	141	6.7
9	209	10.0
10	203	9.7
11	184	8.8
12	195	9.3
Total	2,094	100

The most common causes of the injury were violent assault or nonviolent traumatic injury (*n* = 1,034, 49.4%), slip or fall (*n* = 304, 14.5%), road traffic accidents (*n* = 303, 14.5%), sports (*n* = 236, 11.3%), work-related injuries (*n* = 159, 7.6%) and others (*n*= 5 8, 2.8%) [Table 5]. The most common sports associated with injury was soccer (38.1%).

**Table 5:** Causes of facial bone fractures

*Causes*	*Cases*	*%*
Trauma	1,034	49.4
Violence(‐): injury	556	26.6
Violence(+): assault	478	22.8
Slip or fall	304	14.5
Traffic accident	303	14.5
Sport	236	11.3
Work related	159	7.6
Others	58	2.8
Total	2,094	100

In 60 (2.9%) of the 2,094 patients, the facial fractures were associated with other injuries [Table 6]. Head and neck injuries were the most common isolated injuries associated with facial fractures (13.3%) [Table 6]. Among the patients with injuries to the head and neck area, most had intra cranial injuries with altered levels of consciousness, cervical spine injuries, or optic nerve injury. Some patients (1.3%) had more than one type of associated injury, including other bone fractures [Table 7]. The most common isolated fracture associated with facial fractures was a skull fracture (22.2%) [Table 7]. In 359 (17.1%) of the 2,094 patients, the facial fractures were associated with other soft tissue injuries [Table 8]. The associated injuries were most commonly soft tissue injuries of the face and neck (89.4%) [Table 8].

**Table 6:** Associated injuries

*Associated Injuries*	*Cases*	*%*
Head and neck	5	13.3
Trunk	5	8.3
Lower extremity	4	6.7
Upper extremity	2	3.3
Combined	41	68.3
Total	60	100

**Table 7:** Associated bone fractures

*Associated bone fractures*	*Cases*	*%*
Skull	6	22.2
Upper extremity	5	18.5
Clavicle	4	14.8
Spine	2	7.4
Rib	1	3.7
Lower extremity	1	3.7
Pelvis	1	3.7
Combined	6	22.2
Others	1	3.7
Total	27	100

**Table 8:** Associated soft tissue injuries

*Associated soft tissue injuries*	*Cases*	*%*
Face and neck	321	89.4
Scalp	5	1.4
Upper extremity	3	0.8
Lower extremity	3	0.8
Trunk	1	0.3
Combined	25	7.0
Others	1	0.3
Total	359	100

### Classification of facial bone fractures and treatment

**Figure 1:**
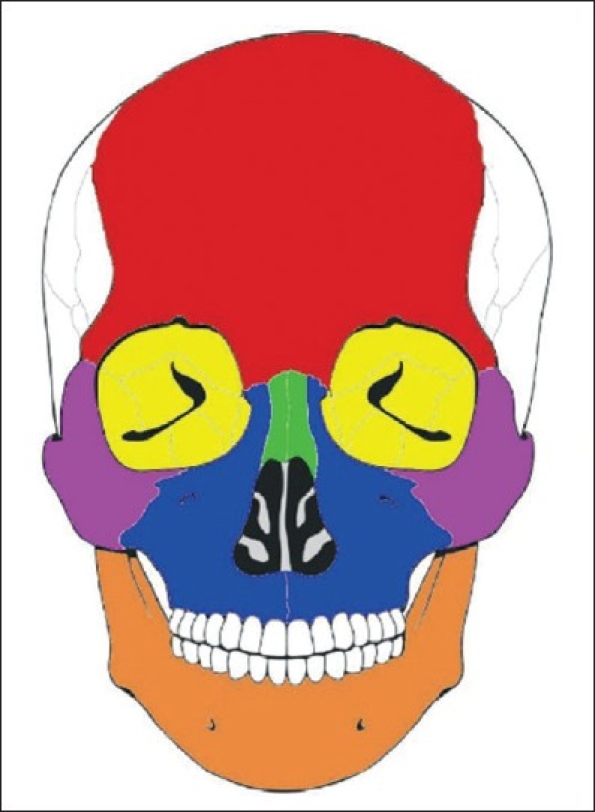
The skeletal region of isolated facial bone fractures: red, frontal bone (0.4%); yellow, orbital bone (9.2%); green, nasal bone (45.9%); blue, maxilla (1.6%); purple, zygoma (6.9%); orange, mandible (35.9%)

**Figure 2:**
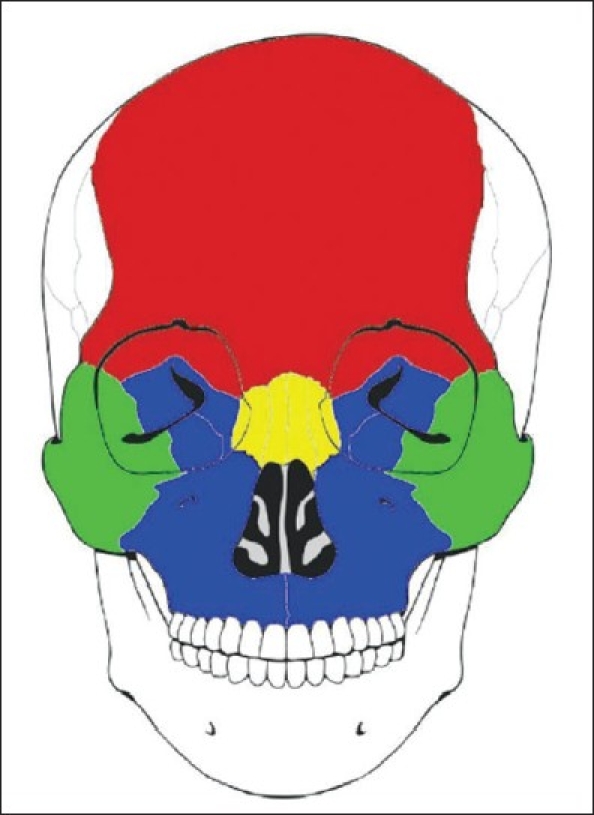
The skeletal region of complex facial bone fractures: red, superior (1.6%); yellow, medial (4.3%); green, lateral (1.1%); blue, inferior (78.3%)

The great majority of cases were isolated injuries (*n* = 1720, 82.1%) [Table 1]. Nasal bone fractures were the most common (*n* = 790, 37.7%), followed by mandible fractures (*n* = 618, 30%) [Table 1, Figure 1]. Tripod fractures were the most common type of complex injuries (*n* = 130, 6.2%) [Table 1]. For complex injuries, the inferior region had the highest frequency of fractures (*n* = 293, 14%) [Table 1, Figure 2].

A closed reduction procedure was performed in 46.3% of the cases, open reduction in 39.7%, closed and open reduction in 6.5% and no surgical intervention in 7.4% [Table 9]. The closed reduction procedure was most commonly performed for nasal bone fractures (93%). Most of the other facial bone fractures were treated by open reduction (39.7%) procedures. The facial bone reduction was carried out on average 5.2 days post injury, and most had surgery within 1 week (72%) [Table 10]. The average hospital stay for the patients with a facial bone fracture was 8.4 days; most were discharged from the hospital within 2 weeks (88.5%) [Table 11].

**Table 9:** Treatment modalities for facial bone fractures

*Treatment modalities*	*Cases*	*%*
Closed reduction	969	46.3
Open reduction	832	39.7
Conservative	156	7.4
Closed and open reduction	137	6.5
Total	2,094	100

**Table 10:** Time interval between accident and surgical treatment for facial bone fractures

*Interval*	*Cases*	*%*
<3 days	449	23.2
<1 week	945	48.8
<2 weeks	485	25.0
<3 weeks	53	2.7
>3 weeks	6	0.3
Total	1,938	100

**Table 11:** Days of hospitalisation

*Weeks*	*Cases*	*%*
<1	449	23.2
1–2	945	48.8
2–3	485	25.0
3–4	53	2.7
>4	6	0.3
Total	1,938	100

For the open reduction procedure, various soft-tissue approaches were used to meet the requirements of adequate fracture exposure and stabilisation at multiple points. The most commonly used approach was the intraoral approach (32.3%), followed by the subcilliary approach (25.3%) [Table 12]. Fracture stabilisation materials for fixation included the miniplate (66%), microplate (21.7%), microplate in combination with the miniplate (9.2%), a resorbable plate (2.4%) and wire (0.8%) [Table 13]. In addition, reconstruction materials included the Medpor (81.5%), a resorbable sheet (16.8%) and bone grafts (1.7%) [Table 14].

**Table 12:** Soft Tissue Approaches

*Soft Tissue Approaches*	*Cases*	*%*
Intraoral	326	32.3
Subciliary	256	25.3
Submandibular	89	8.8
Subciliary and Dingman	82	8.1
Gilles	41	4.1
Through laceration	26	2.6
Riston	25	2.5
Transconjunctival	9	0.9
Dingman	6	0.6
Bicoronal	6	0.6
Combined	142	14.1
Others	2	0.2
Total	1,010	100

**Table 13:** Materials used for fixation

*Materials*	*Cases*	*%*
Miniplate	445	66.0
Microplate	146	21.7
Miniplate and microplate	62	9.2
Resorbable plate	16	2.4
Wire	5	0.7
Others	6	0.9
Total	674	100

**Table 14:** Materials used for reconstruction

*Materials*	*Cases*	*%*
Medpor	97	55.7
Resorbable sheet	20	11.5
Bone graft	2	1.1
Others	5	2.9
Total	119	100

### Complications and prognosis

The complication rate was 6.4% and the most common complication was hypoesthesia (68.4%), followed by diplopia (25.6%) [Table 15]. The average follow-up period for hypoesthesia was 1.2 months and most patients (88%) improved by 1 month, while some (12%) required 3 months for improvement. The average follow-up period for diplopia was 2 months. Most patients improved (61%) during the first month of follow-up and 21% improved by 3 months. Other patients required a longer time to improve (7.1%), while some required 6–12 months.

**Table 15:** Complications associated with facial bone fractures

*Complications*	*Cases*	*%*
Hypoesthesia	91	68.4
Diplopia	34	25.6
Infection	3	2.3
Facial palsy	3	2.3
Haematoma	1	0.8
TMJ ankylosis	1	0.8
Total	133	100

## DISCUSSION

A large number of studies have reported on the aetiology of facial trauma.[1,8] The results of epidemiological investigations vary depending on the demographics of the population studied. Factors such as geographic region, socioeconomic status and temporal factors, including time of year and time of the study, can influence both the type and the frequency of injuries reported for a given population.[5] This makes meaningful comparisons between epidemiological reviews difficult.

The increasing prevalence of facial bone injuries emphasises the necessity for epidemiological surveys to determine optimal prevention strategies and patient management. Such data can inform care-givers the causes and incidences of facial bone fractures. The results of this study showed a high morbidity for facial injuries in the 21–30 years age group followed by the 11–20 years age group. In addition, we found a male predominance among all injuries and ages. Although the annual incidence has increased slightly every year, the monthly frequency was relatively even.

The results of this survey are consistent with prior reports in Korea.[6,7] In general, trauma is primarily a health problem among young men. However, there are differences in the causes of injury by geographic region and socioeconomic status.[1,5] Long-term collection and analysis of epidemiologic data regarding facial fractures in severely injured patients is an important step in the evaluation of conventional preventative measures.[5] It is also necessary to determine trends to help guide the development of new methods of injury prevention.[5] Our results found that violent assault or nonviolent traumatic injuries remain the leading cause of injury. The results of this study suggest that violence prevention programs concentrating on both assault and self-inflicted injury may help decrease the frequency of facial trauma resulting from intentional injuries in this population. In addition, drinking and driving campaigns require strengthening because 30.3% of the all injuries were alcohol-related in our study.

In the present study, the most commonly fractured isolated bones were the nasal bone (37.7%) and the mandible (30%). Our finding agrees with previous studies in Korea.[13,14] This is because the nose is an easy target in personal violence. The most common aetiology of injury in our study is trauma. The nose is projecting, relatively unprotected and with very little soft tissue cover. The most common complex fracture was a tripod fracture (6.2%). The patterns of complex facial bone fractures were classified by the anatomical direction from the orbit. Such fractures can, of course, extend to involve the associated wall of the orbit or may, as in the case of the orbital roof, be an extension from an adjacent area such as the superior rim or the frontal bone. A variety of classifications have been proposed for zygomatic fractures and orbital bone fractures. In 1961, Knight and North classified zygomatic fractures by the direction of displacement on a Waters' view radiograph.[10] They classified 120 fractures into six groups, hypothesising that the stability after reduction might be related to the direction of displacement. This classification has been found to be helpful in predicting the clinical features and necessary treatment, but it does not apply to complex facial bone fractures. Because this system is confined to the zygoma, it does not include the surrounding structures in the classification. In addition, treatment guidelines based on a simple classification of zygomatic fractures was presented by Zingg in 1992.[11] This is a simple classification system for zygomatic fractures based on anatomic points and fracture patterns. However, it is difficult to use one description for different complex facial bone fractures. In 2002, Manolidis analysed orbital bone fractures according to the orbital rim and orbital walls.[12] However, a more accurate classification of injury patterns, including each of these regions, might be achieved by combining the prior classifications into one simple classification to accurately describe the degree of injury to the orbit as a whole and predict the level of surgical intervention required for rigid internal fixation.

Although we may be able to use the available classifications to explain the relationship of the fracture with the surrounding structures, they cannot be applied to all complex facial bone fractures. Therefore, a novel unified classification system for facial injuries is presented here. This proposed complex facial bone fracture classification scheme provides a convenient, descriptive and reproducible method for describing fracture patterns. In our study, the complex fractures were subclassified into five types according to the anatomical direction from the orbit and extension from the adjacent areas, i.e. superior, medial, lateral, inferior and others part. Orbital skeletal injuries are frequently associated with other significant injuries. The orbital rim was considered separately as consisting of four regions, corresponding to the skeletal elements that define it: the frontal (superior), the nasoethmoidal (medial), the zygomatic (lateral) and the maxillary region (inferior). The inferior region was the most frequently involved region in a fracture, occurring in more than three-quarters of the patients (78.3%). This occurs due to its prominent location on the face. The medial region was involved in 4.3%, the superior region in 1.6% and the lateral region in 1.1% of the patients with complex facial bone fractures.

In most of the fractured facial bones, except the nasal bones (*n* = 1,034), an open reduction was performed in 64%, closed reduction in 14%, no surgical intervention in 12% and a closed reduction with open reduction in 11%. The fractured nasal bones accounted for most of the closed reductions (93%) and other facial bones (64%) for open reductions. Our finding is in agreement with previous studies in Korea.[14,15] Facial bone reduction was carried out on average 5.2 days after the injury, when the swelling decreased, and the average hospital stay was 8.4 days. Among the 969 patients treated by open reduction, 674 patients (70%) were treated with one or more internal fixation techniques while 110 patients (12%) were treated with reconstruction methods. The miniplate was the most common osteosynthesis method used (66%)[9] because of the advantages in both the technical requirement and the functional outcome. The functional advantages include rapid improvement and the technical advantages include ease of application, stability and biomechanical compatibility.[1] Medpor was the most commonly used material for reconstruction surgery. Finally, hypoesthesia and diplopia were the most common complications (*n* = 125, 94%). Most patients with these complications improved during the first month (88%) with hypoesthesia and by the third month with diplopia (82%).

## CONCLUSIONS

The findings of this study indicated that epidemiological research of facial fractures allows the presentation patterns of the most affected individuals and the nature of their lesions to be outlined according to the region evaluated. This retrospective study documents the higher risk of fractures in younger males and assaults and other traumas were the commonest causes. Isolated nasal bone fractures were most common. Open reduction was performed in most fractured facial bones, except nasal bones, and hypoesthesia was the most common complication in our study. The insight into the epidemiology of facial bone fractures and associated injuries is useful not only for developing prevention strategies but also for decisions with regard to patient care, development of optimal treatment regimens and appropriate resource allocation. Furthermore, treatment evaluation and complication rate analysis permits a more realistic interpretation of how patients should be managed.
